# Supplementary material for: Multi‐dataset identification of innovative feature genes and molecular mechanisms in keratoconus
Source: J Cell Mol Med. 2024 Sep 19;28(18):e70079. doi: 10.1111/jcmm.70079 (PMC11412914; doi:10.1111/jcmm.70079)
Supplement: Supplementary file 2 — Material S2. [file JCMM-28-e70079-s002.zip › 7.GSEA analysis/5.RNF217-AS1/KEGG_RENIN_ANGIOTENSIN_SYSTEM.html]

Details for gene set KEGG\_RENIN\_ANGIOTENSIN\_SYSTEM[GSEA]

|  || Dataset | RNF217-AS1.group.cls#h\_versus\_l |
| Phenotype | group.cls#h\_versus\_l |
| Upregulated in class | h |
| GeneSet | KEGG\_RENIN\_ANGIOTENSIN\_SYSTEM |
| Enrichment Score (ES) | 0.31817383 |
| Normalized Enrichment Score (NES) | 0.71947527 |
| Nominal p-value | 0.86221296 |
| FDR q-value | 1.0 |
| FWER p-Value | 1.0 |
Table: GSEA Results Summary

  

Fig 1: Enrichment plot: KEGG\_RENIN\_ANGIOTENSIN\_SYSTEM      
 Profile of the Running ES Score & Positions of GeneSet Members on the Rank Ordered List

  

| SYMBOL | TITLE | RANK IN GENE LIST | RANK METRIC SCORE | RUNNING ES | CORE ENRICHMENT || 1 | AGT | na | 1420 | 0.399 | 0.1418 | Yes |
| 2 | AGTR1 | na | 3697 | 0.263 | 0.1967 | Yes |
| 3 | CMA1 | na | 5624 | 0.195 | 0.2303 | Yes |
| 4 | AGTR2 | na | 5655 | 0.194 | 0.3182 | Yes |
| 5 | CTSG | na | 9428 | 0.097 | 0.2537 | No |
| 6 | ENPEP | na | 14183 | 0.029 | 0.1298 | No |
| 7 | MAS1 | na | 14359 | 0.026 | 0.1368 | No |
| 8 | ACE | na | 17438 | -0.006 | 0.0508 | No |
| 9 | CTSA | na | 21264 | -0.043 | -0.0399 | No |
| 10 | LNPEP | na | 21527 | -0.046 | -0.0266 | No |
| 11 | THOP1 | na | 22266 | -0.054 | -0.0233 | No |
| 12 | NLN | na | 22483 | -0.056 | -0.0040 | No |
| 13 | ACE2 | na | 22653 | -0.058 | 0.0175 | No |
| 14 | ANPEP | na | 26864 | -0.123 | -0.0475 | No |
| 15 | MME | na | 27797 | -0.144 | -0.0087 | No |
| 16 | REN | na | 30180 | -0.212 | 0.0198 | No |
| 17 | CPA3 | na | 31026 | -0.243 | 0.1066 | No |
Table: GSEA details [plain text format]

  

Fig 2: KEGG\_RENIN\_ANGIOTENSIN\_SYSTEM      
 Blue-Pink O' Gram in the Space of the Analyzed GeneSet

  

Fig 3: KEGG\_RENIN\_ANGIOTENSIN\_SYSTEM: Random ES distribution      
 Gene set null distribution of ES for **KEGG\_RENIN\_ANGIOTENSIN\_SYSTEM**
